# Supplementary material for: Study of Synthesis Pathways of the Essential Polyunsaturated Fatty Acid 20:5n-3 in the Diatom Chaetoceros Muelleri Using 13C-Isotope Labeling
Source: Biomolecules. 2020 May 21;10(5):797. doi: 10.3390/biom10050797 (PMC7277837; doi:10.3390/biom10050797)

**SUPPLEMENTARY DATA - GC-FID CHROMATOGRAMS**

The identification of the peaks detected for *Chaetoceros muelleri* FAME samples has been conducted using commercially-available individual purified standards and home-made standard mixtures (developed along years) by comparing their retention times to those of the samples considered, on both columns.

Among the most used standards for the FA identification in aquatic samples, we used the Supelco® 37 Component FAME Mix, a comprehensive mixture of 37 FAMEs ranging from 4:0 to 24:1n-9, as well as PUFAs No.1 and PUFAs No.3 from Sigma-Aldrich, complex qualitative standard mixtures extracted from Menhaden oil. Only Supelco® 37 is used as a quantitative standard to control FAME response factors while PUFAs No.1 and PUFAs No.3 are only used qualitatively. In addition to this approach and whenever necessary, the identification of FAMEs was confirmed using GC-mass spectrometry (GC-MS). Peak identification was achieved using commercial spectral libraries (NIST 11), the LipidWeb website (available in: https://www.lipidhome.co.uk, Christie, 2020) or via home-made FA mass spectral libraries.

Quantification of FAME is made using the peak areas. Absolute concentrations are determined by adding an internal standard (tricosanoic acid 23:0, noted C23:0 in the following chromatograms).

Following chromatograms show the different fatty acids that have been identified in this study using SUPELCO® 37 standard (in black) and PUFAs mixtures (PUFAs No.1 – in red, and PUFAs No.3 – in green) on both polar column (DB-WAX: 30 mm x 0.25 mm ID x 0.25 µm, Agilent) and apolar column (DB-5: 30 m x 0.25 mm ID x 0.25 µm, Agilent) as well as for both studied fractions (NL: neutral lipids, PL: polar lipids). Each time, the full chromatogram is presented at two scales (a and b) to validate peak separation and shape for quantification.

Fatty acids notation: **X:Yn-Z** (X: number of carbons, Y: number of double bonds, Z: position of the first double bond starting from the methyl-end of the acyl chain). Fatty acids configuration – t: *trans*.

**SUPELCO 37 – DB-5 (05.06.2018) [a] scale 80000 µV [b] scale 20000 µV**


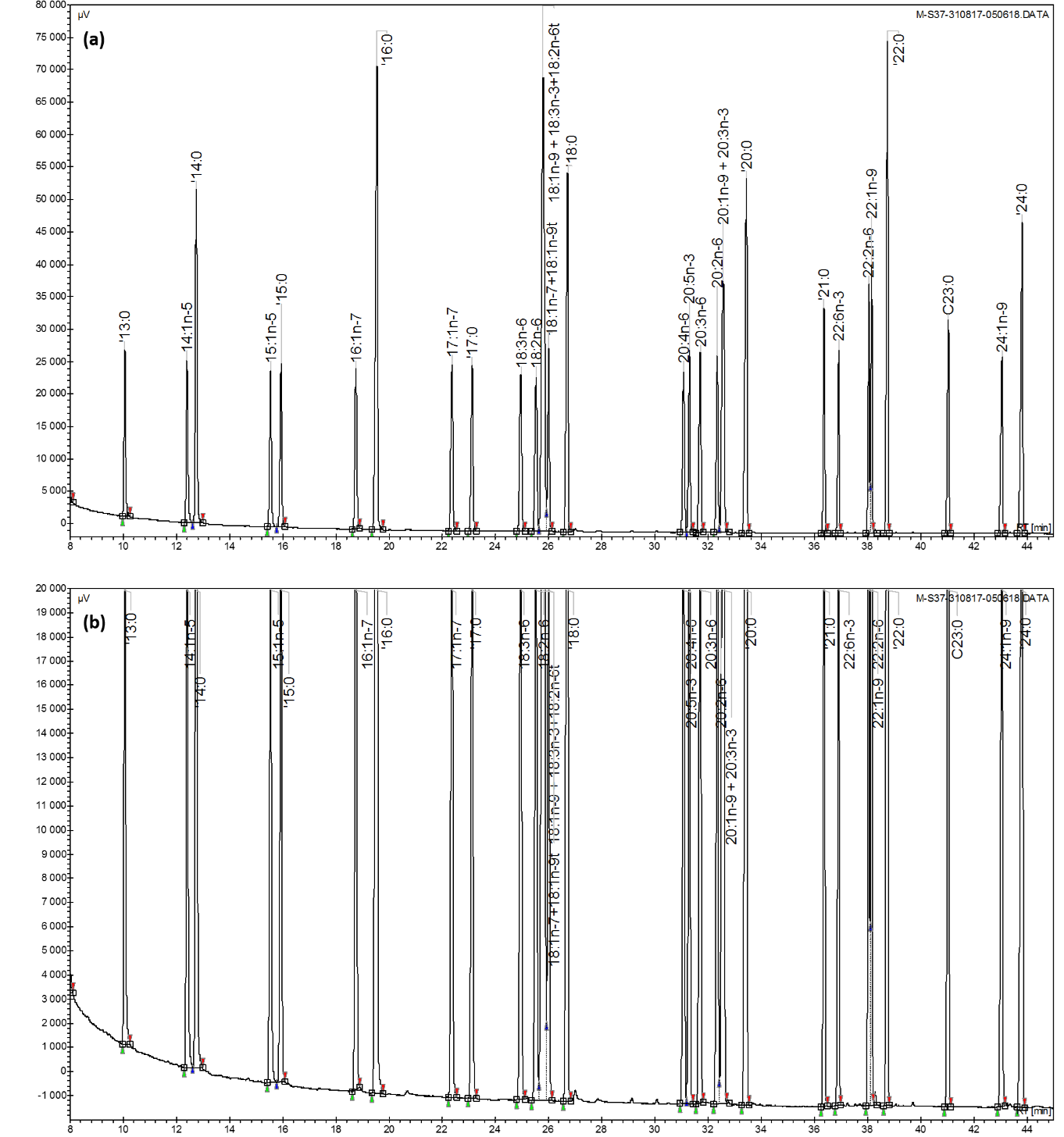


**SUPELCO 37 – DB-5 (03.08.2018) [a] scale 80000 µV [b] scale 20000 µV**


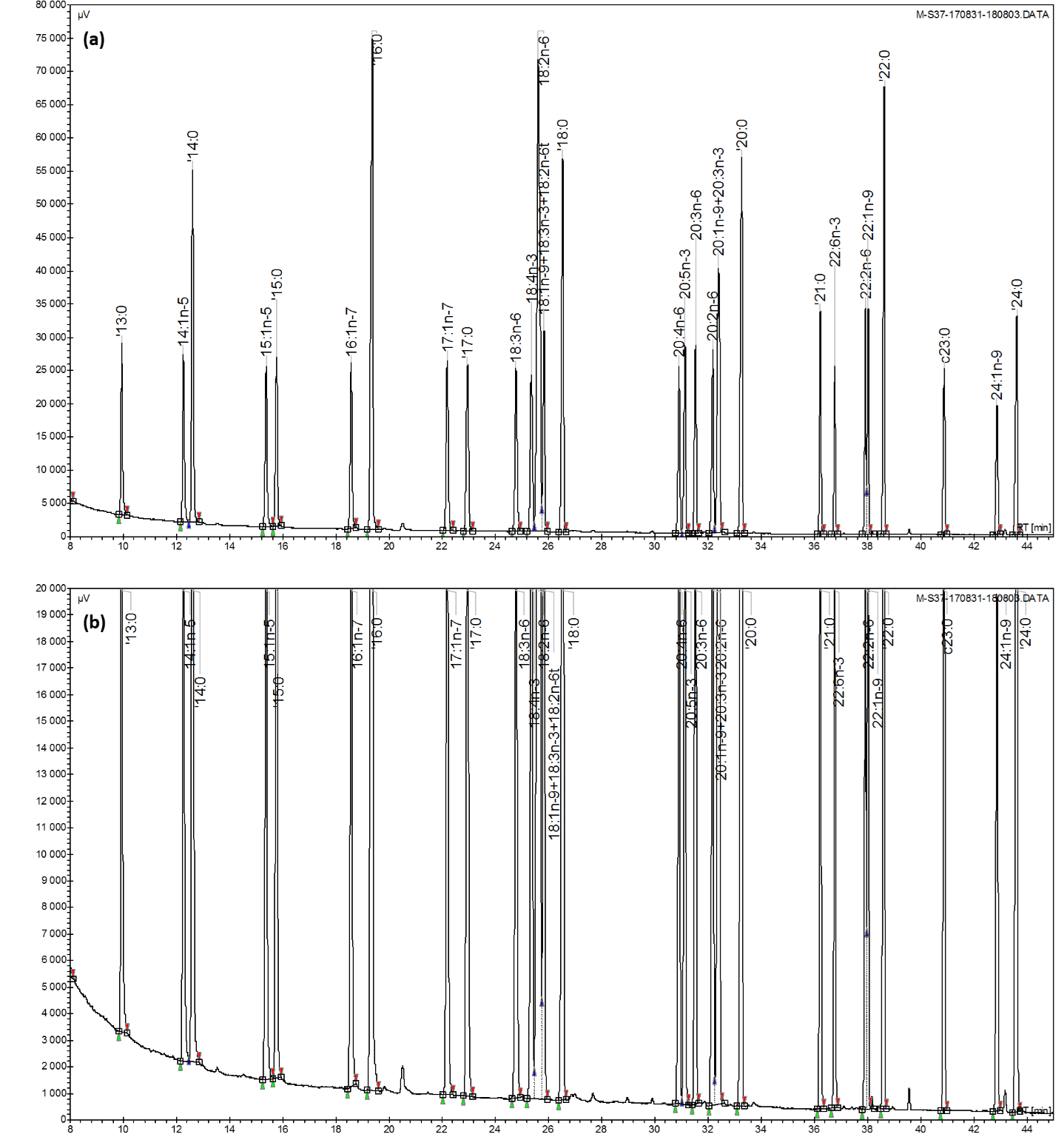


**PUFAs No.1 – DB-5 (05.06.2018) [a] scale 110000 µV [b] scale 20000 µV**


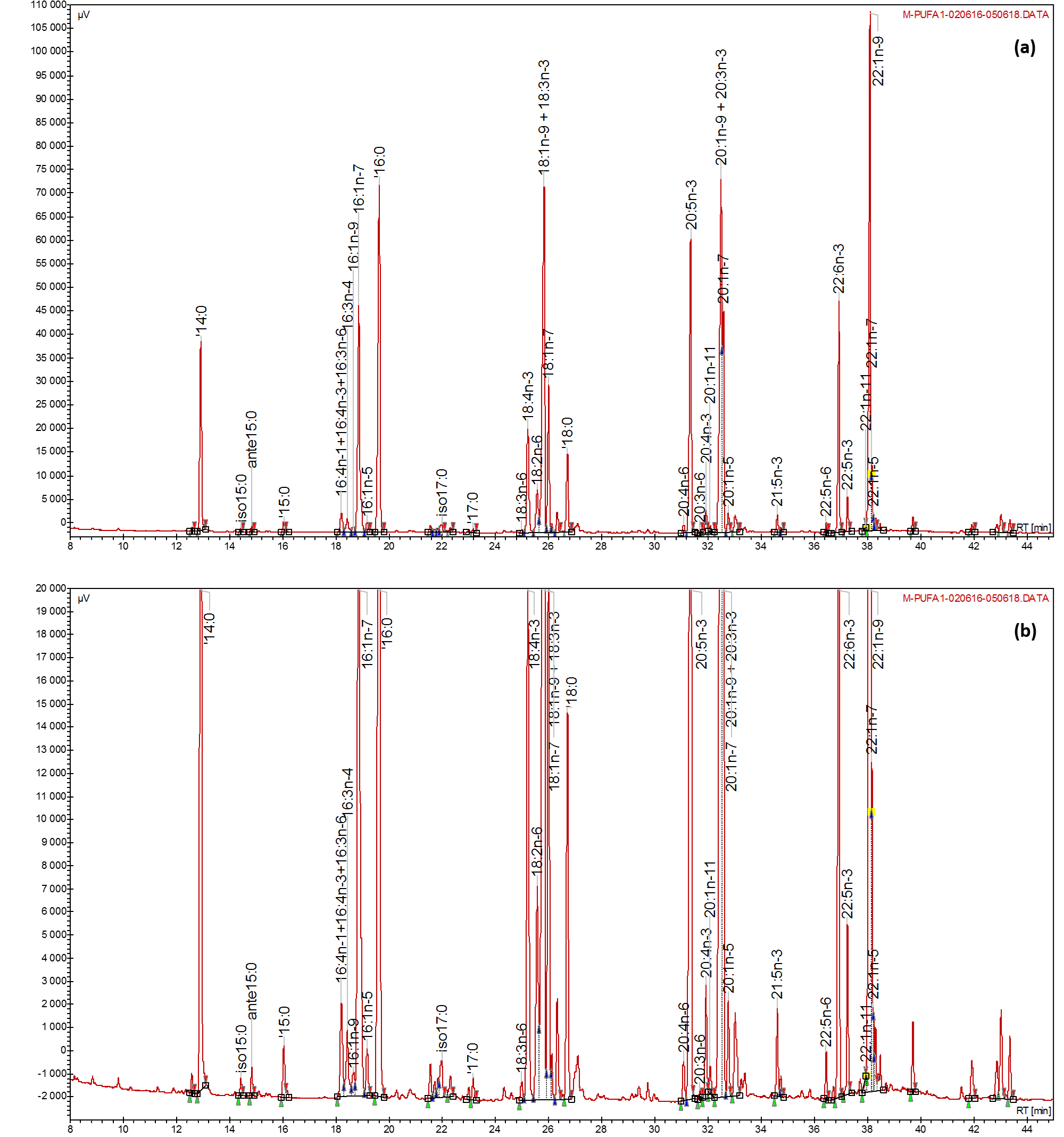


**PUFAs No.1 – DB-5 (03.08.2018) [a] scale 100000 µV [b] scale 20000 µV**


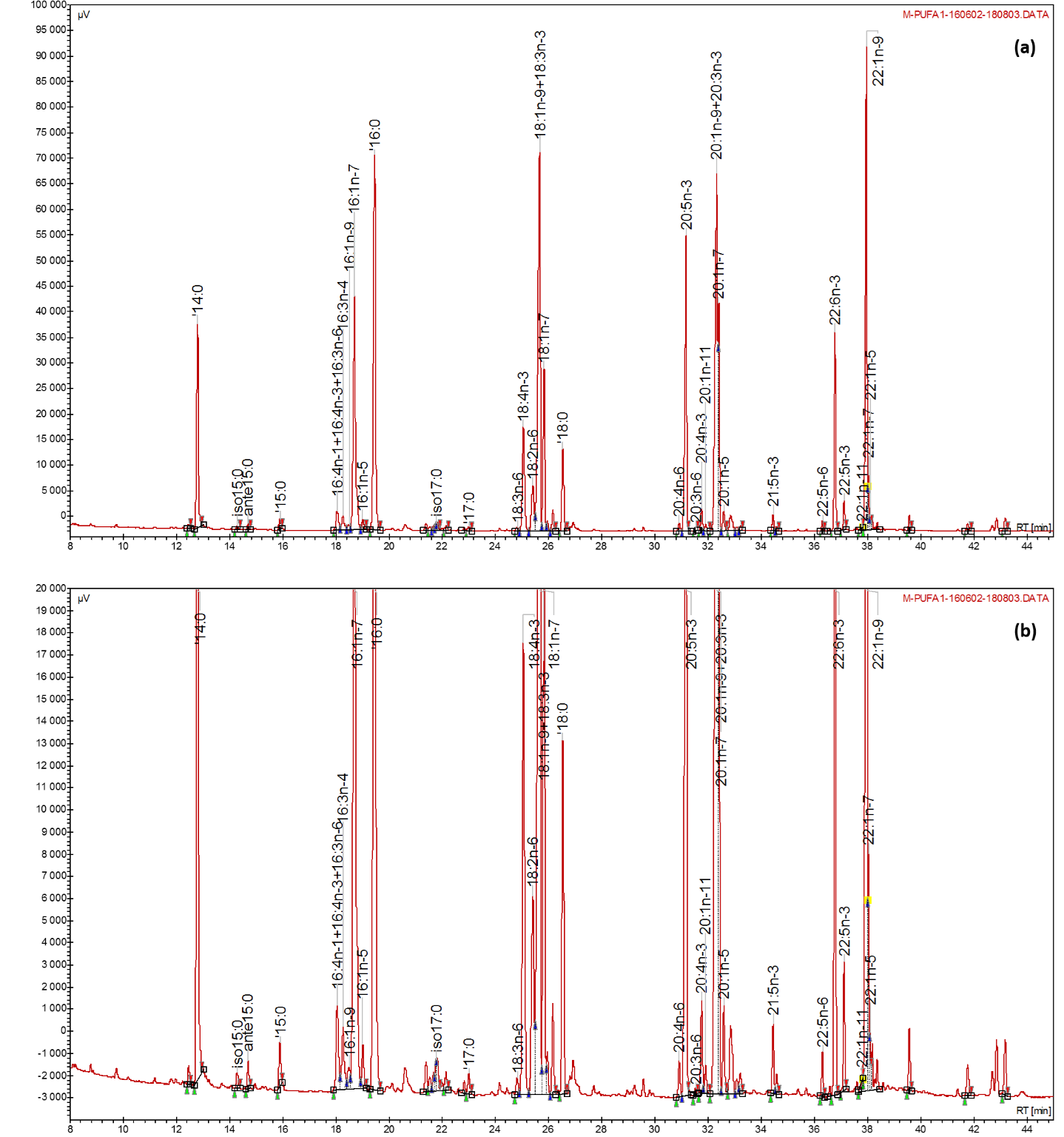


**PUFAs No.3 – DB-5 (05.06.2018) [a] scale 110000 µV [b] scale 20000 µV**


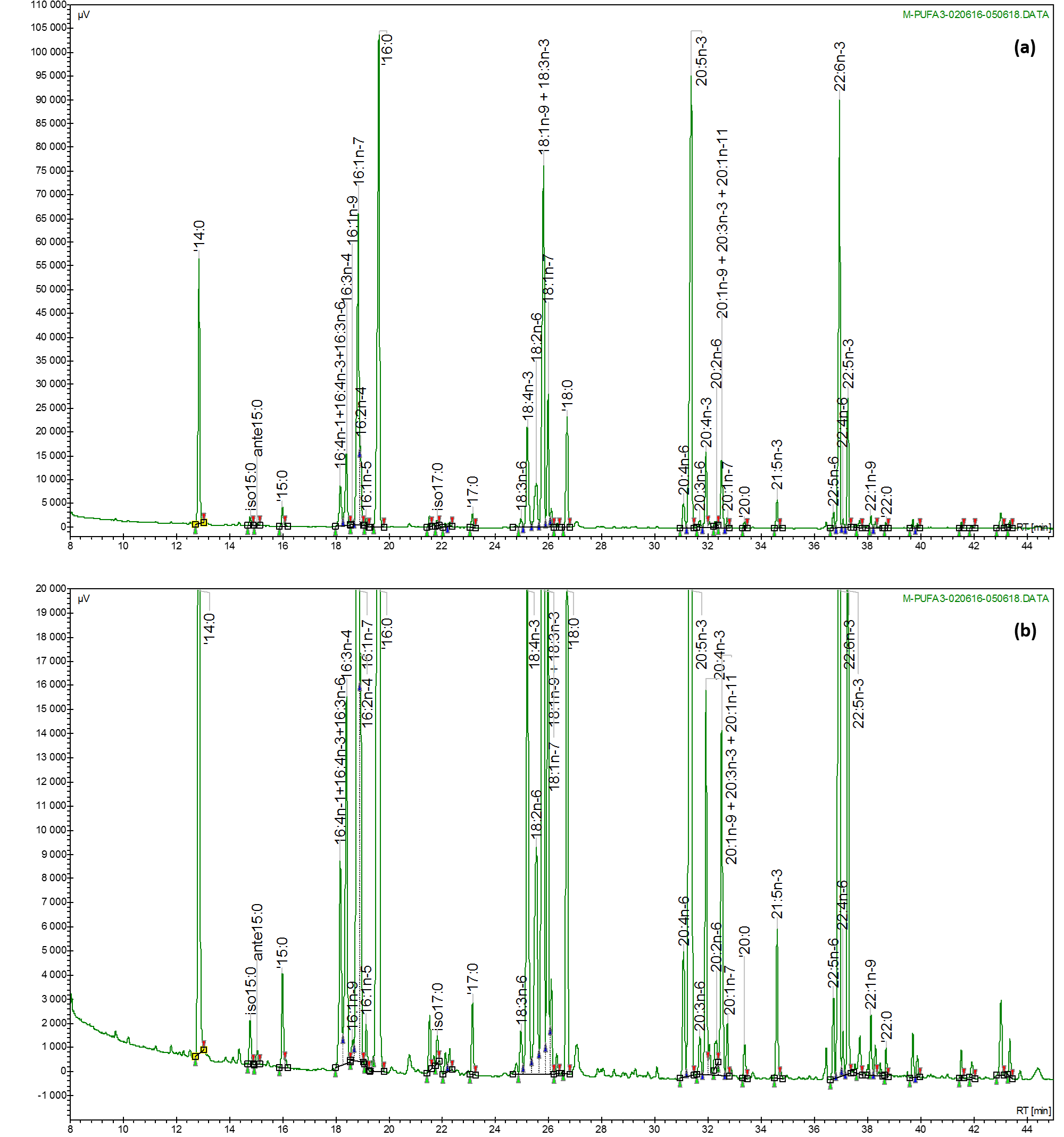


**PUFAs No.3 – DB-5 (03.08.2018) [a] scale 110000 µV [b] scale 20000 µV**


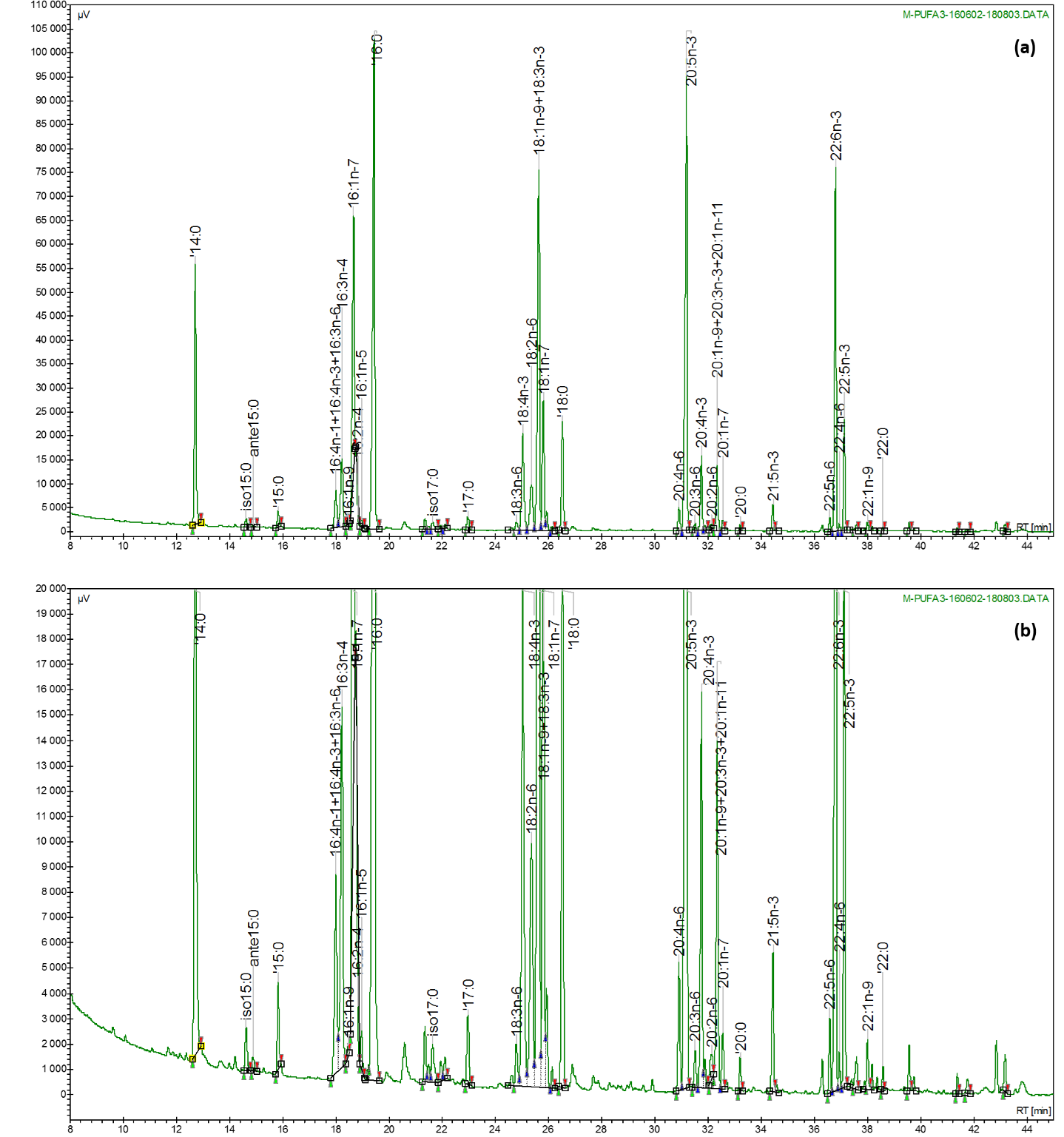


**SAMPLE *Chaetoceros muelleri* Neutral Lipid (NL)
DB-5 [a] scale 110000 µV [b] scale 20000 µV**


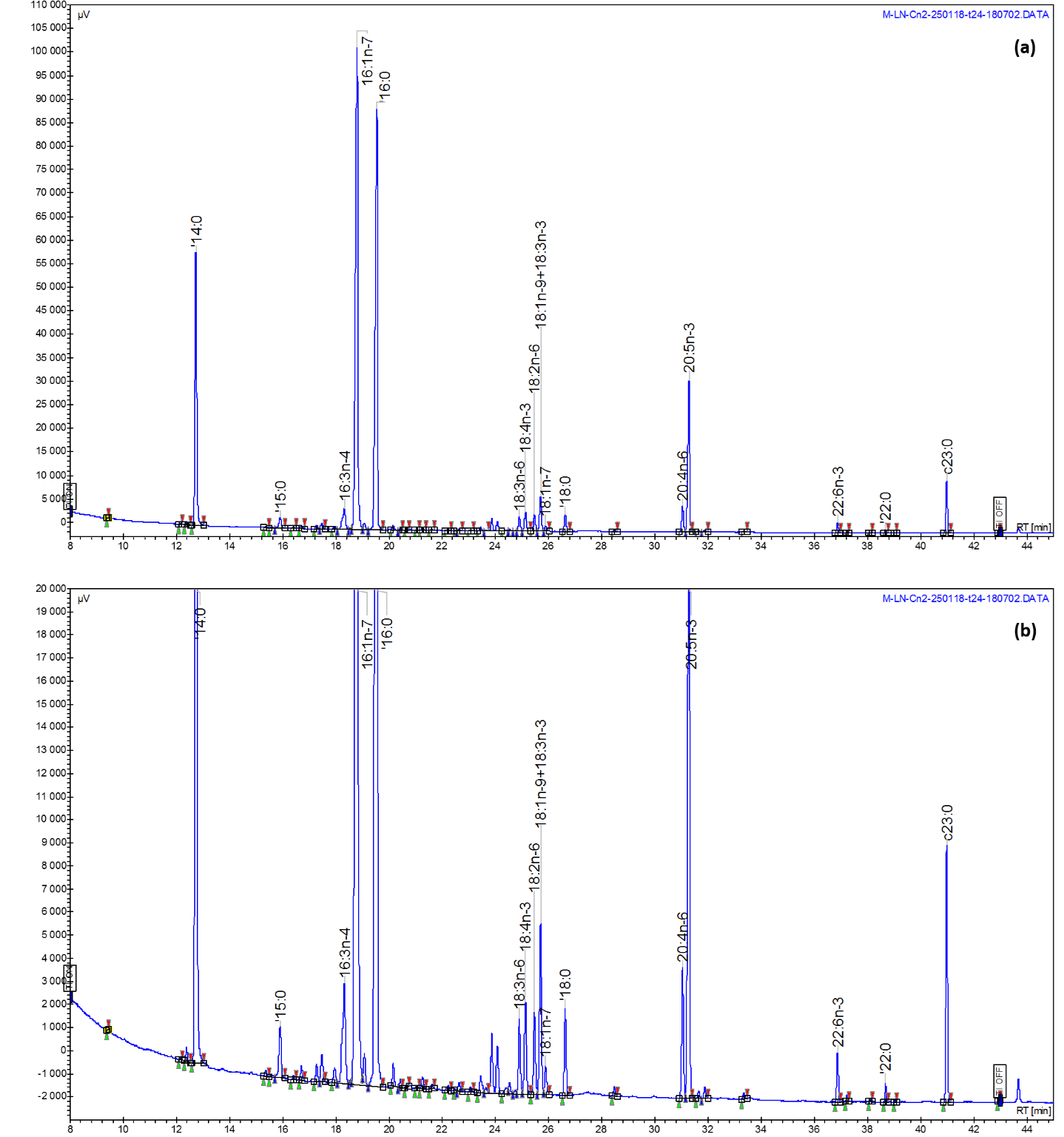


**SAMPLE *Chaetoceros muelleri* Polar Lipid (PL)
DB-5 [a] scale 90000 µV [b] scale 20000 µV**


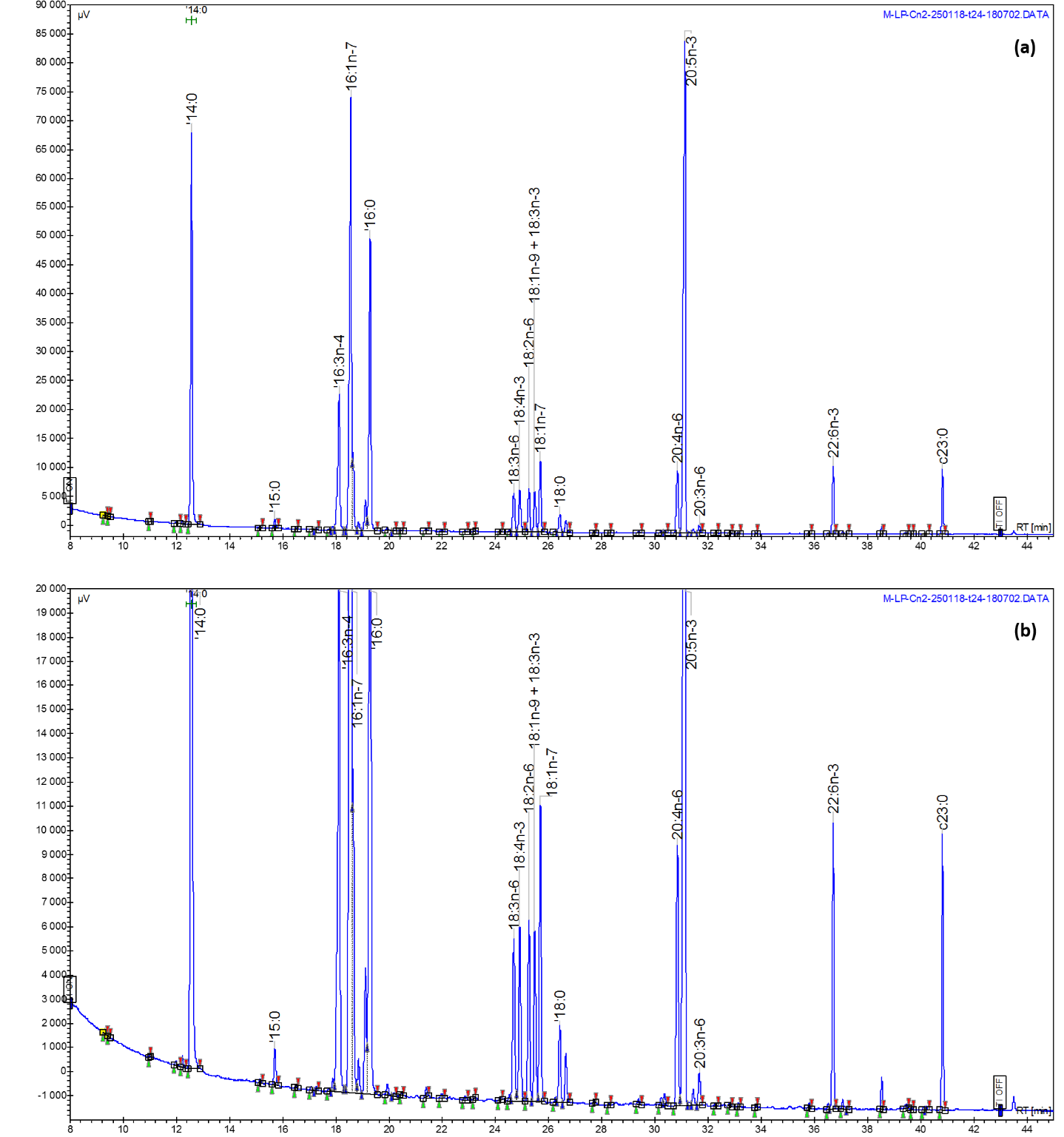


**SUPELCO 37 – DB-WAX [a] scale 80000 µV [b] scale 20000 µV**


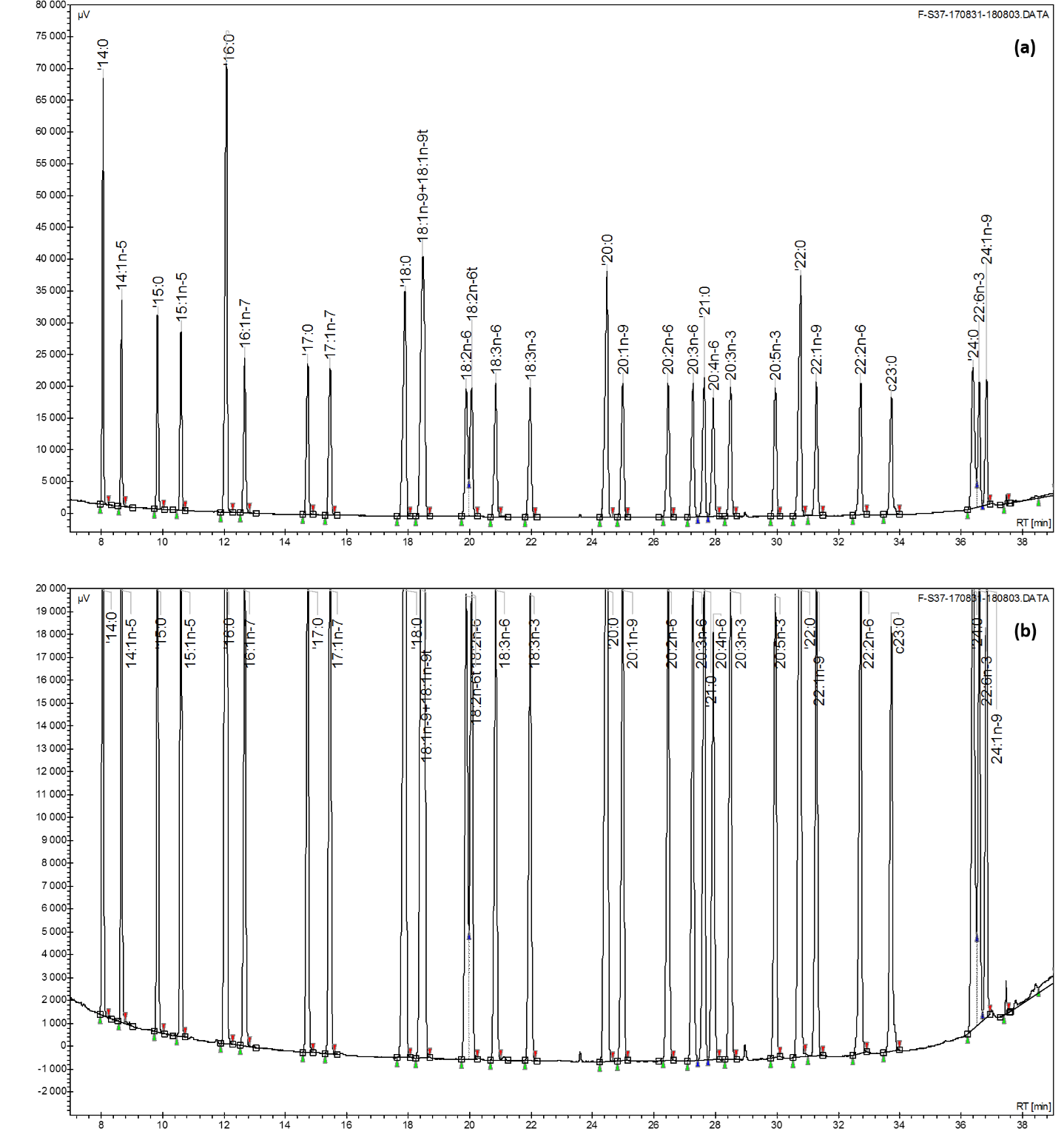


**PUFAs No.1 – DB-WAX [a] scale 80000 µV [b] scale 20000 µV**


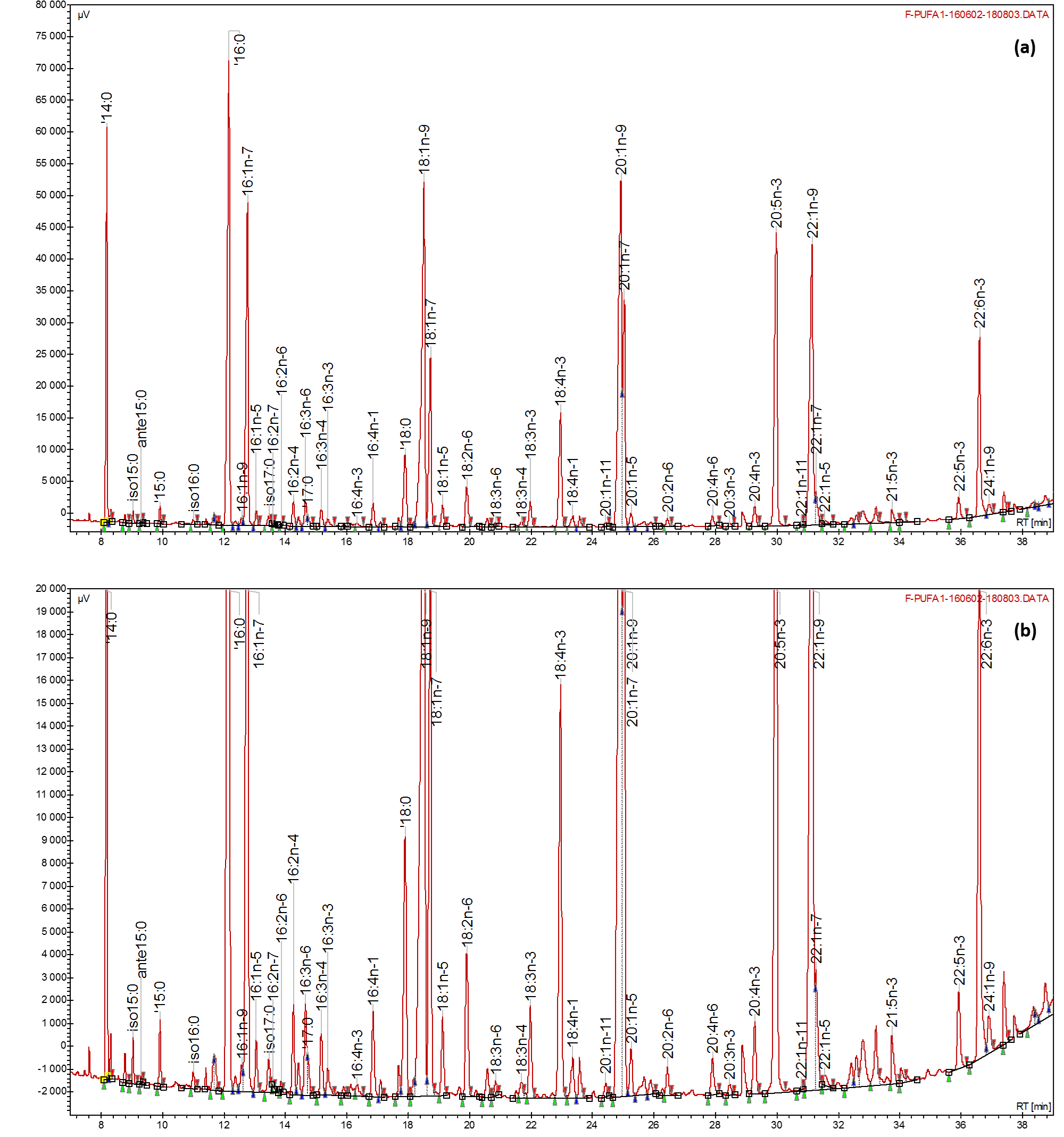


**PUFAs No.3 – DB-WAX [a] scale 100000 µV [b] scale 20000 µV**


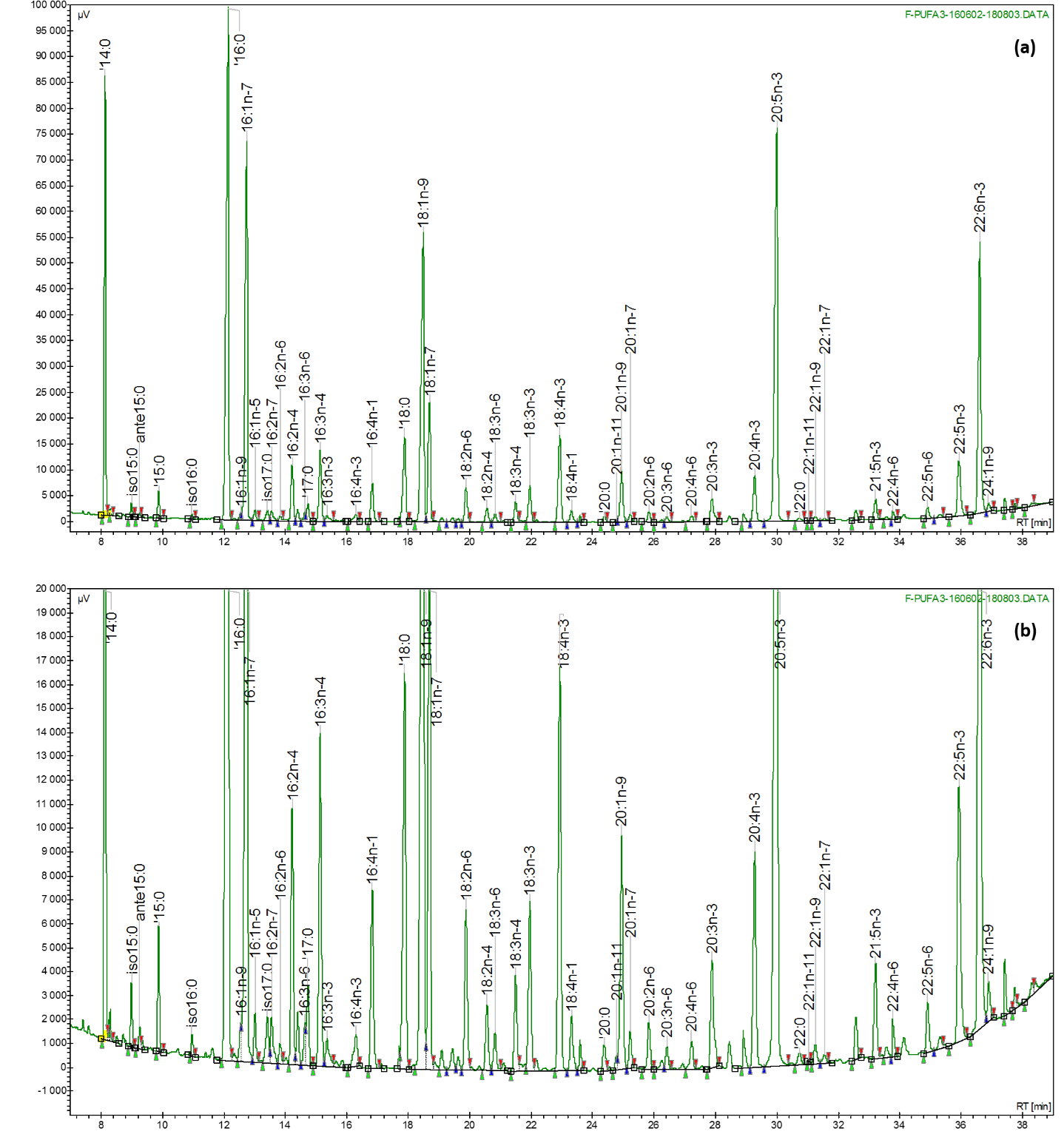


**SAMPLE *Chaetoceros muelleri* Polar Lipid (PL)
DB-WAX [a] scale 110000 µV [b] scale 20000 µV**


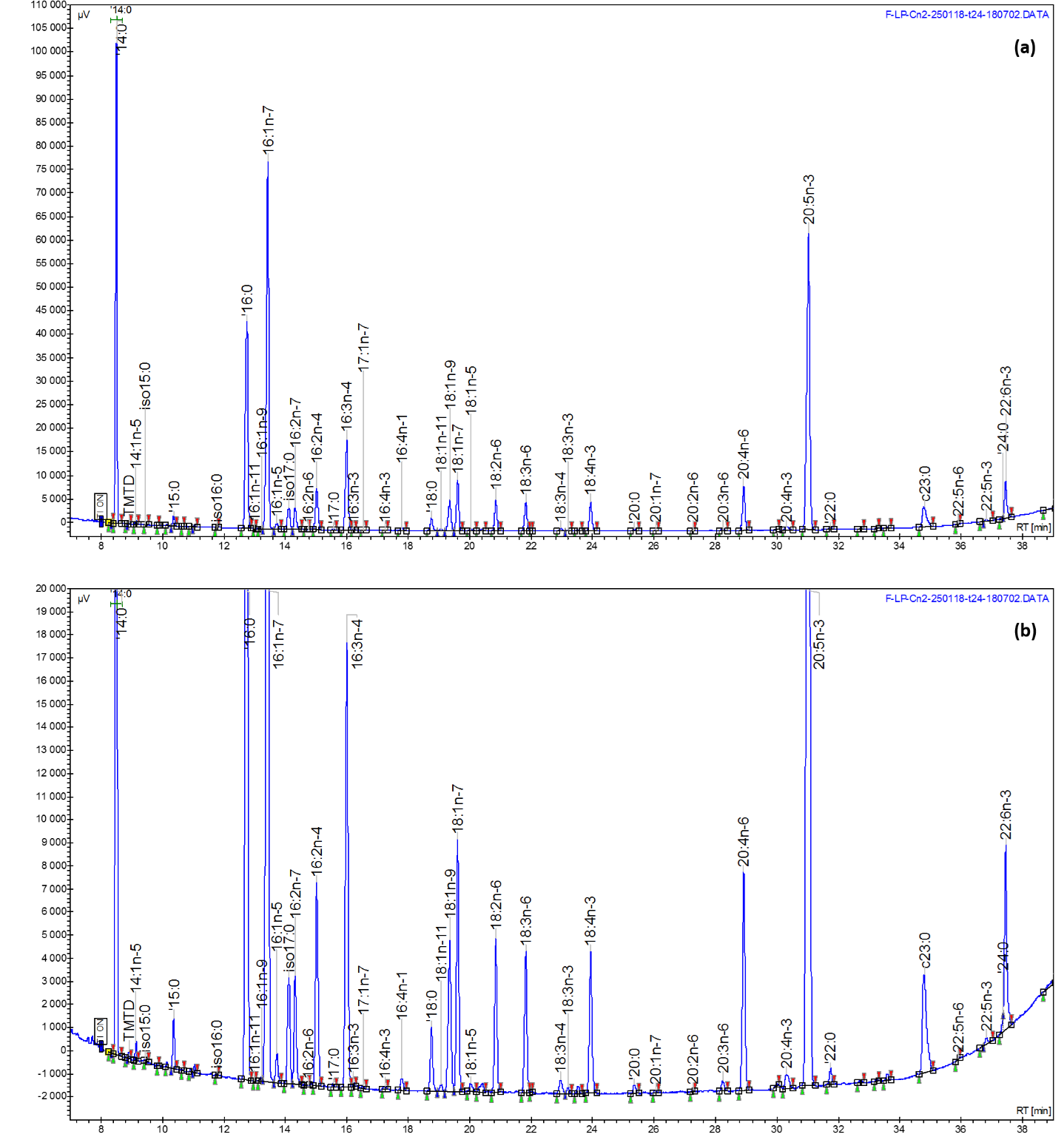


**SAMPLE *Chaetoceros muelleri* Neutral Lipid (NL)
DB-WAX [a] scale 110000 µV [b] scale 20000 µV**


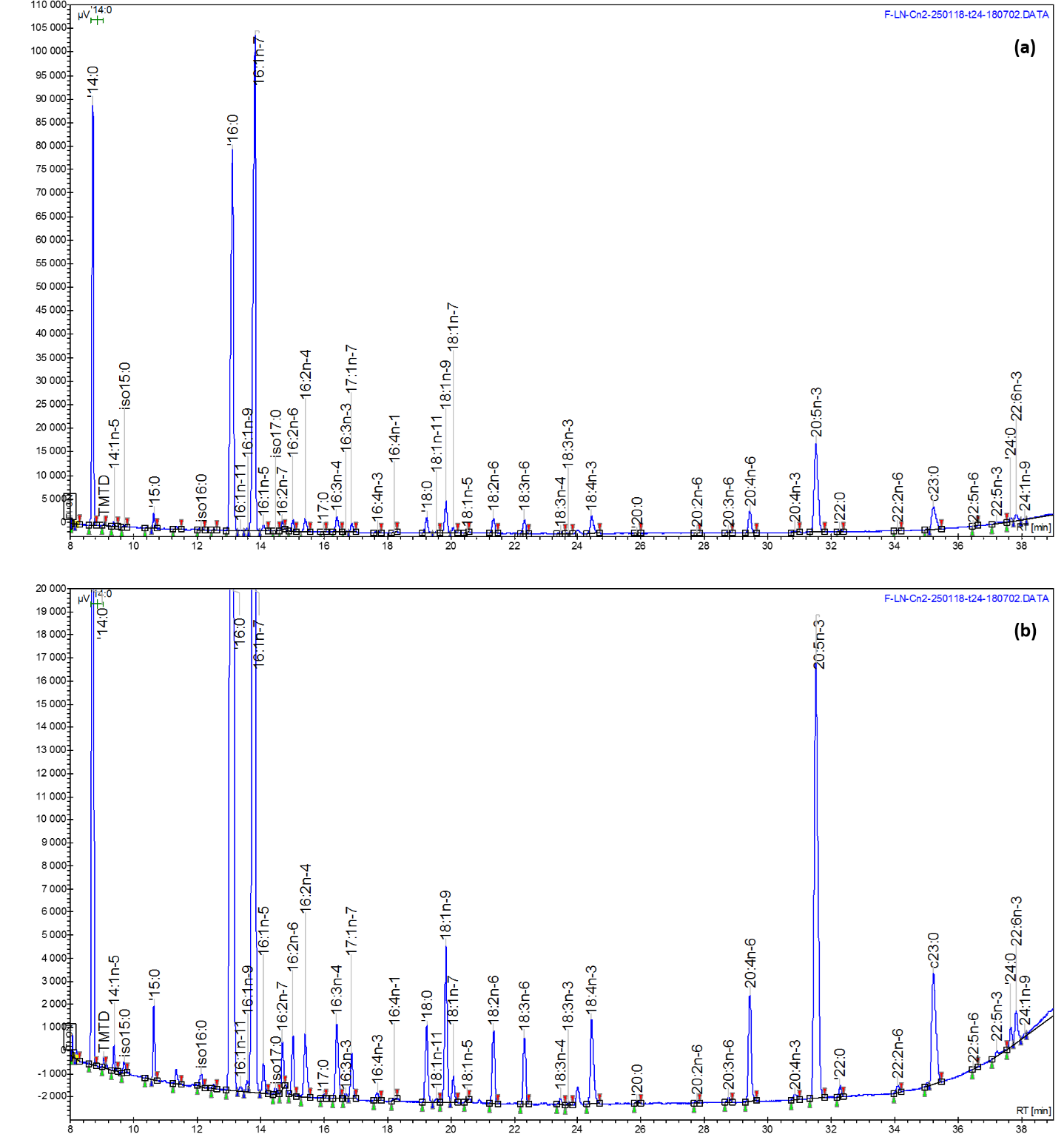

Supplement: Supplementary file 1 [file biomolecules-10-00797-s001.zip › Remize et al_Supplementary Data.docx]
